# Stabilization of a recalcitrant and aggressive necrobiotic xanthogranuloma with tofacitinib^☆^

**DOI:** 10.1016/j.abd.2025.501159

**Published:** 2025-06-28

**Authors:** Miguel Mansilla-Polo, Carlos Abril-Pérez, Daniel Martín-Torregrosa, Rafael Botella-Estrada

**Affiliations:** a Department of Dermatology, Hospital Universitario y Politécnico La Fe, Valencia, Spain; b Instituto de Investigación Sanitaria (IIS) La Fe, Valencia, Spain; c Department of Dermatology, Facultat de Medicina i Odontología, Universitat de València, Valencia, Spain

*Dear Editor,*

Necrobiotic Xanthogranuloma (NXG) is a form of non-Langerhans histiocytosis characterized by the development of yellow-orange infiltrated papules and plaques, with the most common location being the periorbital area. Occasionally, there is extracutaneous involvement, including ocular, respiratory, cardiac, or cerebral. Up to 80% of cases are associated with monoclonal gammopathy and other neoplasms.^1^ The treatment of NXG is complex and data on therapeutic options are limited, making the best approach to treatment unclear. We describe the case of a patient with refractory NXG who experienced an adequate response to tofacitinib, a non-selective inhibitor of Janus Kinases (JAK), which preferentially blocks JAK1 and JAK3.

A 54-year-old woman presented to the Department of Dermatology with refractory NXG. Specifically, the lesions had appeared at 18‒20 years of age (>30 years of evolution), and throughout her life, she had been treated with topical, intralesional, and systemic corticosteroids, methotrexate, multiple surgeries, CO_2_ laser, hydroxychloroquine, extracorporeal photochemotherapy, rituximab, Intravenous Immunoglobulins (IVIG), and cyclophosphamide. Despite the latter treatment (cyclophosphamide), combined with systemic corticosteroids, the clinical condition was progressive. There was no other cutaneous or systemic involvement, and laboratory tests, including protein electrophoresis, were normal. On examination (Fig. 1), yellowish plaques of bilateral palpebral and periocular involvement were observed, predominantly on the left side. The lesions had a shiny surface and a slightly erythematous surrounding edge. They were hard, deeply adherent, and caused some visual restriction in the left eye. The latest biopsy (Fig. 2) and all previous ones were compatible with NXG with deep involvement (muscular invasion), also confirmed by ultrasound and Magnetic Resonance Imaging (MRI). Given the refractory clinical condition, active lesions, and multiple previous treatments, it was decided by a multidisciplinary team to initiate tofacitinib therapy, initially at a dose of 5 mg/12 hours, and due to good tolerance, the dose was increased to 10 mg/12 hours after 3 months. After one year of treatment (Fig. 3), the lesions had stabilized, and even the size of some of them had reduced, especially the left upper eyelid, where the visual restriction had improved. They were also less evident on palpation, and the surrounding rim was less erythematous. The lesions remained the same at the 18-month review. Tolerability during the 1.5 years of treatment was adequate, except for an upper respiratory condition that required discontinuation of treatment for a few days at 10 months of treatment, during which time he reported worsening of the lesions. There were no other adverse effects, and follow-up laboratory tests were normal. On follow-up brain MRI at 1.5 years, the lesions remained stable. Given the adequate therapeutic response, it was decided to continue treatment.

**Fig. 1:**
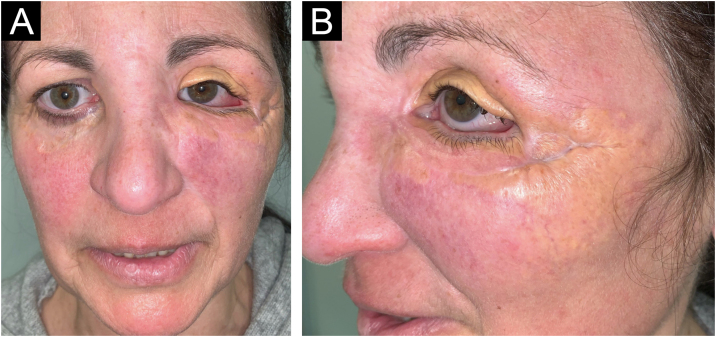
Clinical presentation of the lesions before starting tofacitinib. Yellowish, shiny, and infiltrated plaques located around the eyelids and periocular region bilaterally, with more pronounced involvement in the left eye, where they caused visual restriction. Surrounding erythema around the plaques.

**Fig. 2:**
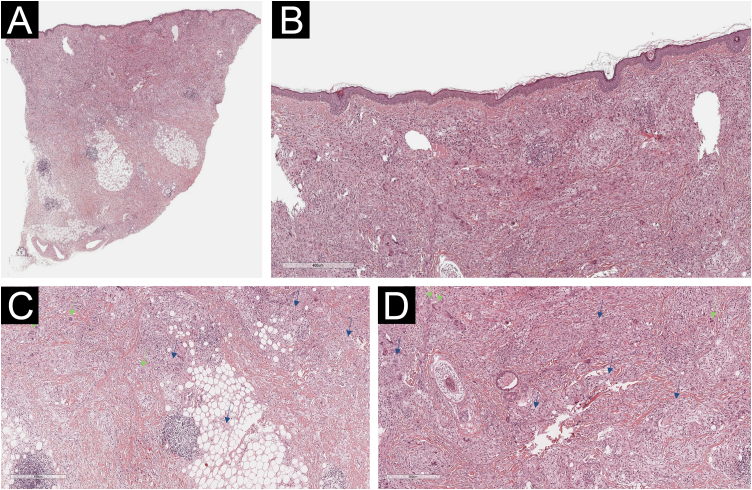
Histological examination of the left lower eyelid lesion (Hematoxylin & eosin; panel A 20×, panel B 80×, panel C 140×, panel D 160×). Histology showed collagenous fibrous tissue in the dermis, interspersed with increased lymphohistiocytic cellularity with abundant foamy histiocytes (blue arrows in panels C and D) and multinucleated giant cells (green arrows in panels C and D).

**Fig. 3:**
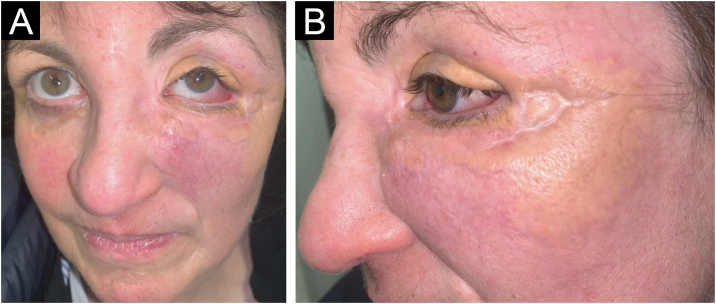
Clinical presentation of the lesions one year after starting tofacitinib. Improvement of the lesions, with a slightly smaller diameter, less active border, and improvement in left visual restriction. Similarly, upon palpation, the lesions felt less infiltrated.

The etiology of NXG, which is thought to be related to altered macrophage lipid homeostasis, has not yet been elucidated. Recently, ABCG5/G8 variants have been identified that encode heterodimers containing transporter and transmembrane domains essential for reverse cholesterol transport and sterol clearance.^1^ The accumulation of lipid particles can lead to the upregulation of pro-inflammatory cytokines such as Interleukin (IL) 6 and tumor necrosis factor-alpha. Animal studies have shown that JAK inhibitors can prevent IL-6 trans-signaling, which may explain the efficacy of tofacitinib on NXG in our patients.^2^ Similarly, the JAK pathway has been reported to play a role in the maintenance of granulomas. Thus, JAK inhibition may promote granuloma resolution, as has been described in other granulomatous diseases such as granuloma annulare^3^ and sarcoidosis.^3^, ^4^ On the other hand, treatment of NXG is challenging and the best approach to treatment is uncertain. Randomized clinical trials are lacking, so most recommendations are based on studies with limited evidence. Before starting treatment, it is essential to rule out monoclonal gammopathy and associated malignancy.^5^, ^6^ Generally, the first-line therapy consists of alkylating agents such as chlorambucil, cyclophosphamide, or melphalan, administered with or without systemic glucocorticoids. Other treatments used include IVIG, thalidomide, lenalidomide, interferon α-2a, plasmapheresis, dapsone, antimalarials, phototherapy, azathioprine, methotrexate, or rituximab. For localized lesions, local treatments such as surgery or CO_2_ laser therapy can be employed.^5^, ^6^, ^7^ Our patient had undergone most of these treatments and still had refractory lesions with progressive growth. For this reason, and based on the reported efficacy of tofacitinib in isolated cases in granulomatous diseases, including histiocytosis such as necrobiotic xanthogranuloma^2^ and multicentric reticulohistiocytosis,^8^ and non-histiocytosis such as granuloma annulare^3^ or sarcoidosis,^3^, ^4^ it was decided to initiate treatment with tofacitinib. Although caution must be exercised when drawing conclusions from the treatment of isolated cases, the efficacy of tofacitinib observed in our patient, a refractory case, suggests it is a therapeutic option for such NXG cases. However, it should be noted that JAK inhibitors have been associated with numerous side effects, including infections (especially herpes zoster, respiratory infections), cardiovascular events (thrombosis, infarction), hematological changes, and a possible increased risk of neoplasia.^9^, ^10^ Therefore, their use should always be justified, and safer therapeutic alternatives should be offered to the patient if available.

## Financial support

None declared.

## Authors’ contributions

Miguel Mansilla-Polo: Managed clinical treatment and procedures; contributed to the development of this paper, had access to the data, and played a role in writing this manuscript.

Carlos Abril-Pérez: Managed clinical treatment and procedures; contributed to the development of this paper, had access to the data, and played a role in writing this manuscript.

Daniel Martín-Torregrosa: Managed clinical treatment and procedures; contributed to the development of this paper, had access to the data, and played a role in writing this manuscript.

Rafael Botella-Estrada: Supervised the work, had access to the data, and played a role in writing this manuscript.

## Conflicts of interest

None declared.
